# Melanoma-specific mutation hotspots in distal, non-coding, promoter-interacting regions implicate novel candidate driver genes

**DOI:** 10.1038/s41416-024-02870-w

**Published:** 2024-10-04

**Authors:** Michael Pudjihartono, Nicholas Pudjihartono, Justin M. O’Sullivan, William Schierding

**Affiliations:** 1 Liggins Institute, The University of Auckland, Auckland, New Zealand; 2 The Maurice Wilkins Centre, The University of Auckland, Auckland, New Zealand; 3 Australian Parkinson’s Mission, Garvan Institute of Medical Research, Sydney, NSW Australia; 4 MRC Lifecourse Epidemiology Unit, University of Southampton, Southampton, UK; 5 Singapore Institute for Clinical Sciences, Agency for Science, Technology and Research (A*STAR), Singapore, Singapore; 6 Department of Ophthalmology, University of Auckland, Auckland, New Zealand

**Keywords:** Genetics research, Melanoma

## Abstract

**Background:**

To develop targeted treatments, it is crucial to identify the full spectrum of genetic drivers in melanoma, including those in non-coding regions. However, recent efforts to explore non-coding regions have primarily focused on gene-adjacent elements such as promoters and non-coding RNAs, leaving intergenic distal regulatory elements largely unexplored.

**Methods:**

We used Hi-C chromatin contact data from melanoma cells to map distal, non-coding, promoter-interacting regulatory elements genome-wide in melanoma. Using this “promoter-interaction network”, alongside whole-genome sequence and gene expression data from the Pan Cancer Analysis of Whole Genomes, we developed multivariate linear regression models to identify distal somatic mutation hotspots that affect promoter activity.

**Results:**

We identified eight recurrently mutated hotspots that are novel, melanoma-specific, located in promoter-interacting distal regulatory elements, alter transcription factor binding motifs, and affect the expression of genes (e.g., *HSPB7*, *CLDN1*, *ADCY9* and *FDXR*) previously implicated as tumour suppressors/oncogenes in various cancers.

**Conclusions:**

Our study suggests additional non-coding drivers beyond the well-characterised *TERT* promoter in melanoma, offering new insights into the disruption of complex regulatory networks by non-coding mutations that may contribute to melanoma development. Furthermore, our study provides a framework for integrating multiple levels of biological data to uncover cancer-specific non-coding drivers.

## Background

Skin cancers rank as the most prevalent cancers worldwide [1], with melanoma being the deadliest amongst them [2]. To develop targeted treatments, it is crucial to identify the entire spectrum of sites that, when mutated, are causative of melanoma development. However, a large fraction of mutations in cancer genomes are passengers, which do not confer a detectable selective advantage to cells and, therefore, do not undergo positive selection [3]. Conversely, regional recurrence of somatic mutations is indicative of the positive selection process that marks driver events, contributing to tumorigenesis [4].

Large-scale cancer sequencing projects such as The Cancer Genome Atlas (TCGA) [5] and the International Cancer Genome Consortium (ICGC) [6] have initially focused on the coding regions, which led to the identification of hundreds of driver mutations in protein-coding genes [7]. In contrast, few non-coding drivers are currently known. Thus far, the most characterised non-coding driver is located in the promoter of the telomerase reverse transcriptase (*TERT*) gene, which upregulates *TERT* expression in melanoma and many other cancer types [8–10]. It is hypothesised that many other non-coding drivers exist outside the *TERT* promoter. And so, there has been a shift in interest to systematically analyse the non-coding regions, which represent 98% of the human genome where the vast majority of somatic mutations reside [11], to identify potential driver mutations [12]. However, most large-scale efforts have concentrated on gene-adjacent non-coding elements (e.g., promoters and non-coding RNAs [9, 13–17]) leaving intergenic distal regulatory elements largely unexplored.

Due to their role in regulating gene expression, distal regulatory elements represent a promising group of loci within the non-coding genome within which to investigate driver mutations. For example, recurrent mutations in distal regulatory elements of *PAX5* [18] and *TAL1* [19] have been identified in leukaemia. However, these elements often regulate non-adjacent genes over large genomic distances [20–23], making it challenging to systematically identify their true target genes based on linear genomic sequences. Hi-C technologies have emerged as valuable tools to identify long-range interactions within the 3D organisation of the genome [24]. We have previously shown that such long-range connections can identify targets of germline gene regulation [25–27]. However, such genome organisation varies across tissue types [28, 29], underscoring the need to analyse data from tissues relevant to the disease state.

This study leveraged melanoma-specific Hi-C data to map distal regulatory elements that physically interact with the promoter of 18,044 protein-coding genes genome-wide in melanoma. We then integrated these data with whole-genome sequence (WGS) and gene expression data from the TCGA/ICGC Pan-Cancer Analysis of Whole Genomes (PCAWG) to develop multivariate linear regression models, identifying distal somatic mutation hotspots that impact promoter activity. This approach identified eight candidate recurrently mutated non-coding drivers that are melanoma-specific, located in promoter-interacting distal regulatory elements, alter transcription factor binding motifs, and affect the expression of genes (e.g., *HSPB7*, *CLDN1, ADCY9* and *FDXR*) previously implicated as tumour suppressors/oncogenes in various cancers. In summary, our study integrates multiple levels of biological data to uncover novel melanoma-specific non-coding drivers, providing a solid foundation for targeted functional validations.

## Results

### Construction and characterisation of a promoter-interaction network in melanoma

**Fig. 1:**
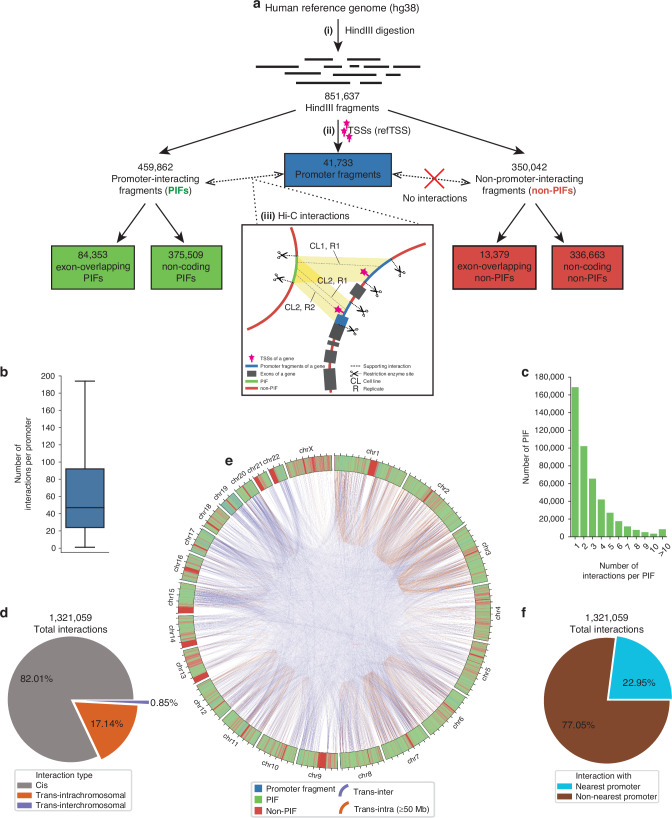
Workflow for identification of 375,509 non-coding, promoter-interacting fragments in melanoma-specific Hi-C cell-lines. **a** The workflow consisted of three parts: (i) Digestion of the human genome into non-overlapping genomic fragments; (ii) Identification of promoter fragments; (iii) Identification of distal fragments interacting with the promoter fragment(s) of each protein-coding gene in the genome. In the cartoon example, a fragment-promoter interaction has a total of 3 supporting interactions from 3 different replicates of 2 different cell lines. The pipeline classified the total genomic fragments into distinct fragment classes (red, blue, and green boxes). **b** Number of interactions per promoter with distal promoter-interacting fragments (PIFs). **c** Number of interactions per PIF with promoters. **d** Proportion of different distance classes of interactions in relation to the total unique PIF interactions. **e** Circos plot illustrating the distribution of fragment classes across the genome and the interactions anchored at gene promoters. For clarity, only trans-inter and a subset of trans-intrachromosomal (≥50 Mb) interactions are shown. **f** Proportions of interactions involving nearest promoters and those involving non-nearest promoters.

The hg38 human reference genome was *in-silico* digested at HindIII restriction enzyme cleavage sites (A/AGCTT), resulting in 851,637 non-overlapping genomic fragments [Fig. 1a(i), Supplementary Table 1], with a median length 2219 bp [Supplementary Fig. 1a]. A total of 41,733 unique genomic fragments overlapped a promoter region harbouring active TSS [30] [Fig. 1a(ii), Supplementary Table 2], defined as “promoter fragments” [Supplementary Table 3].

To catalogue the putative regulatory elements specific to melanoma, we mapped all physical interactions between the promoter fragment(s) of each gene and the rest of the genome using Hi-C data from the two ENCODE melanoma cell lines (SK-MEL-5 and RPMI7951 [31]) [Fig. 1a(iii)]. This promoter-interaction network [Supplementary File 1] contained 459,862 unique non-promoter distal fragments that interacted with at least one gene promoter, referred to as “promoter-interacting fragments” (PIFs). The majority of these PIFs (*n* = 375,509, 82%) were located within the non-coding regions of the genome (termed “non-coding PIFs”) [Fig. 1a].

We observed that over 98% of gene promoters interacted with at least one PIF, with a median of 47 interactions per promoter [Fig. 1b]. Conversely, most PIFs interacted with one or two gene promoters [Fig. 1c]. These interactions primarily occurred within a distance of ≤1 Mb (*Cis*, 82% of the total interactions), while 17% occurred over distances >1 Mb (*Trans-intrachromosomal*), with the remaining 1% involved interactions between chromosomes (*Trans-interchromosomal*) [Fig. 1d]. The distribution of fragment classes across the genome and the interactions anchored at gene promoters is summarised on [Fig. 1e].

We then investigated whether PIFs predominantly interacted with the nearest promoter. Our analysis revealed that 77% of PIF interactions occurred with a non-nearest promoter [Fig. 1f]. This highlights the intricate and long-range spatial arrangements that potentially contribute to transcriptional regulation.

### Melanoma promoter-interacting fragments are under stronger purifying selection

**Fig. 2:**
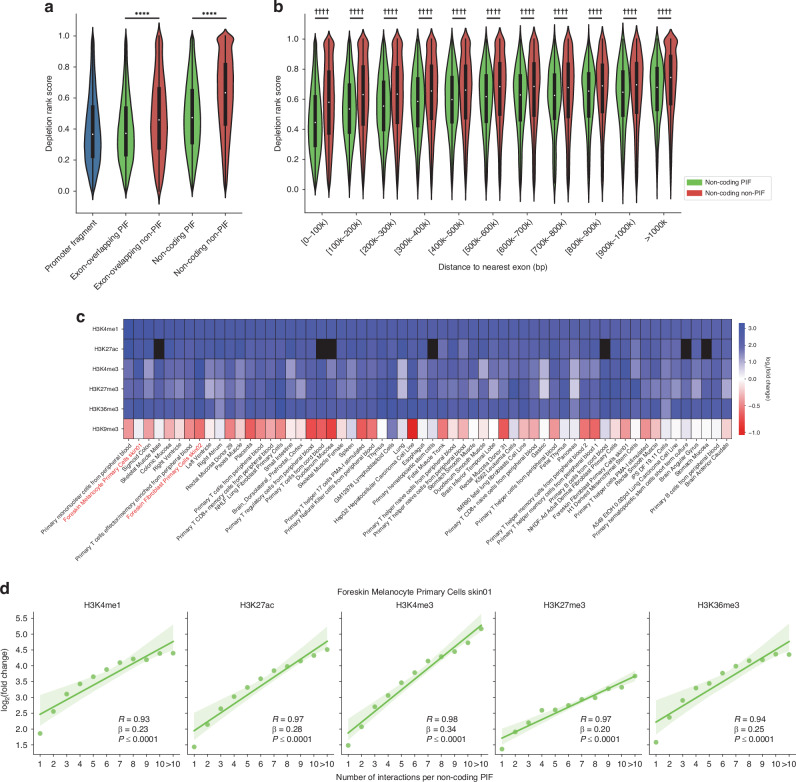
Promoter-interacting fragments (PIFs) are enriched for markers of active regulatory sequences. **a** Both exon-overlapping and non-coding PIFs are subject to increased purifying selection compared to their non-PIF counterparts, as shown by their significantly lower depletion rank (DR) score. The Wilcoxon rank-sum test was used to accommodate deviations from normality. Significance levels: *P ≤ 0.05, **P ≤ 0.01, ***P ≤ 0.001, ****P ≤ 0.0001. **b** Non-coding PIFs consistently exhibited significantly lower DR scores than non-coding non-PIFs, regardless of their proximity to coding exons. Statistical significance was determined using the Wilcoxon rank-sum test with Bonferroni correction for multiple comparisons. Significance levels: †*adj. P* ≤ 0.05, ††*adj. P* ≤ 0.01, †††*adj. P* ≤ 0.001, ††††*adj. P* ≤ 0.0001. **c** Enrichment of non-coding PIFs for histone marks across tissues and cell lines. Cell lines are sorted based on the fold change of H3K4me1. The top 60 cell lines with the highest H3K4me1 fold change are displayed. Black cells represent cell lines with no available data in the Roadmap Epigenomics project. **d** For non-coding PIF, an increased number of promoter interactions correlates with higher levels of regulatory histone marks in melanocyte. The dots represent individual data points, each corresponding to a specific count of interacting promoters of non-coding PIFs and the associated log_2_(fold change) of a histone mark. The green line represents the linear regression fit, with the shaded region indicating the 95% confidence interval for the regression line. The Pearson correlation coefficient quantifies the strength and direction of the linear relationship, while the slope (β) indicates the change in log_2_(fold change) per unit increase in promoter interactions. The linear regression *P* value (*P*) assesses the significance of the relationship.

To investigate the potential regulatory roles of the identified PIFs, we examined whether these PIFs showed evidence of purifying selection indicative of active regulatory sequences [32]. We employed the depletion rank (DR) score, a measure of genome-wide sequence conservation based on the UK biobank WGS data, where lower DR scores indicate regions under stronger sequence constraints [33]. Our analysis demonstrated that both exon-overlapping and non-coding PIFs exhibited significantly lower DR scores compared to their respective non-PIF counterparts [Fig. 2a].

We observed that non-coding PIFs tended to be located in closer proximity to exons compared to non-coding non-PIFs [Supplementary Fig. 1b]; at the same time, the DR score of fragments displayed an increasing trend with increasing distance from exons [Supplementary Fig. 1c]. To ascertain whether the lower DR scores of non-coding PIFs relative to non-coding non-PIFs could be entirely attributed to the latter being situated further from exons, we evaluated the DR scores within distinct distance groups. Our findings indicated that distance from exon correlated with DR score for non-coding PIFs, but that distance was not the sole factor impacting the difference in DR score, as significant differences remained across every distance group [Fig. 2b]. Therefore, the enrichment of lower DR scores in non-coding PIFs potentially reflects their regulatory characteristics, regardless of their proximity to coding exons.

### Melanoma promoter-interacting fragments are enriched for skin-specific histone marks of gene regulation

Across 127 cell lines and tissues, non-coding PIFs were enriched in histone marks associated with enhancers and regulatory elements, with melanocyte and skin fibroblast tissues among those exhibiting strong enrichments [Fig. 2c, Supplementary Table 4]. By contrast, H3K9me3—a histone mark associated with inactive heterochromatin [34]—was depleted across tissues. Additionally, a strong positive correlation (Pearson correlation coefficient) was observed between the number of promoter interactions of non-coding PIFs and the enrichment of regulatory histone marks in melanocyte [Fig. 2d]. Therefore, our finding is consistent with the potential transcriptional regulatory roles of the identified PIFs.

### Melanoma promoter-interacting fragments harbour melanoma-specific non-coding mutation hotspots

**Fig. 3:**
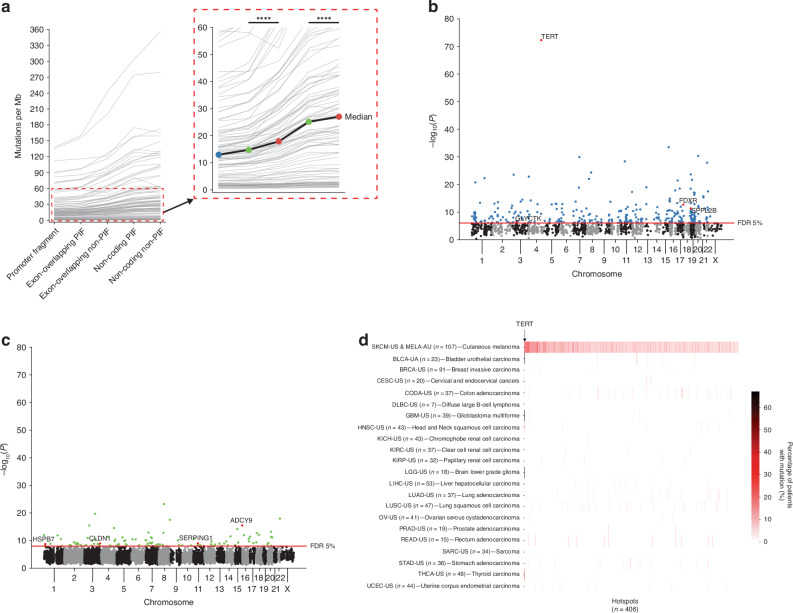
Non-coding somatic mutation hotspots in melanoma are melanoma-specific. **a** Promoter-interacting fragments (PIFs) have significantly depleted somatic mutation rates as compared to non-PIFs in the melanoma cohort of PCAWG. Grey lines represent data on individual patients (n = 107). The black line shows the median mutation rate across patients. Statistical significance was assessed using Wilcoxon signed-rank test. Significance levels: *P ≤ 0.05, **P ≤ 0.01, ***P ≤ 0.001, ****P ≤ 0.0001. Manhattan plots of somatic mutation hotspots in (**b**) promoter fragments and (**c**) non-coding PIFs. Red dots indicate hotspots identified as significant (FDR ≤ 5%) somatic eQTLs in the melanoma-only and/or extended analyses [Fig. 4a, b]. **d** Heatmap showing the percentage of patients (n = 871) in each of the 22 cancer types with mutations in each melanoma hotspot (n = 406). Hotspots are sorted according to their *adj. P* values. Aside from *TERT*, most hotspots are melanoma-specific, leading to most areas of the plot having 0% non-melanoma patients with mutations (70% of the identified hotspots are exclusively mutated in melanoma alone).

To investigate the impacts of somatic mutations within PIFs, we analysed somatic single-nucleotide variations (SNVs) using WGS data from 107 melanoma patients in the PCAWG consortium. This cohort included 37 patients from the TCGA SKCM-US project and 70 from the ICGC MELA-AU project. Promoter fragments showed the lowest mutation rate among all fragment classes in this melanoma cohort [Fig. 3a]. Importantly, exon-overlapping and non-coding PIFs exhibited significantly lower mutation rates compared to their respective non-PIF counterparts [Fig. 3a]. This aligns with prior findings suggesting that functional regulatory regions tend to have reduced somatic mutation rates in cancers, possibly due to selective constraints or chromatin accessibility factors [17, 35].

The recurrence of somatic mutations above the expected background rate in specific genomic regions is suggestive of a positive selection process which may indicate driver events during tumour evolution [4]. To identify potentially functional non-coding somatic mutations, we utilised MutSpot [36] to detect regions with such recurrence, termed “hotspots”, in promoter fragments and non-coding PIFs. To further account for melanoma-specific mutational biases [37, 38], we incorporated melanoma-specific epigenetic and sequence features alongside the standard MutSpot features into the selection of genomic features for the construction background mutation models (methods) [Supplementary Fig. 2a, b].

Overall, the *TERT* promoter constituted the most significant hotspot in our analysis (*P* = 7.3 × 10^−73^) [Fig. 3b]. However, beyond this well-documented hotspot [8–10], we also identified 303 additional significant (FDR ≤ 0.05) hotspots in promoter fragments [Fig. 3b, Supplementary Table 5a] and a further 102 in non-coding PIFs [Fig. 3c, Supplementary Table 5b], totalling 406 significant hotspots. The number of mutated hotspots in each patient correlated with their mutation load [Supplementary Fig. 3]. These hotspots are relatively short, with a median length of 41 bp and a maximum length of 114 bp. Of note, despite their lower mutation rate, promoter fragments harboured threefold more significant hotspots than non-coding PIFs and exhibit a lower *P* value overall [Fig. 3b, c]. There was no significant relationship between the mutation status of target genes and their associated hotspot(s) for the vast majority (93%) of cases, suggesting that these mutations may occur independently [Supplementary Table 6, Supplementary Fig. 4].

Cancer-associated germline single nucleotide polymorphisms (SNPs) identified by genome-wide association studies (GWAS) tend to reside in tissue or cancer type-specific regulatory elements [39–41] and are implicated in modulating the activity of these elements [42]. To investigate whether the identified somatic mutation hotspots were pan-cancer or melanoma-specific, we analysed the WGS data of an additional 764 patients across 21 cancer types from the TCGA portion of PCAWG (totalling 871 patients across 22 cancer types). Beyond a relatively small number of hotspots (e.g., the *TERT* promoter, which is known to be highly mutated across cancer types [8–10]) the majority of the identified hotspots (n = 281, 70%) are exclusively mutated in melanoma alone [Fig. 3c]. This melanoma specificity likely arises from a combination of factors, including the use of melanoma Hi-C cell lines, which may have captured lineage-specific functional elements, and melanoma’s unique mutational landscape, characterised by a high burden of UV-induced mutations.

### Melanoma-specific non-coding mutation hotspots alter the expression of eight candidate driver genes

The impact of a mutation on relevant biological functions can suggest its likelihood of being a driver [43]. Non-coding driver mutations should, therefore, act as distal regulators of gene expression. Changes at a locus that lead to changes in target gene expression are known as expression quantitative trait loci (eQTLs), which in germline contexts are often leveraged to infer the functional implications of non-coding GWAS risk SNPs [44]. We sought to determine whether the somatic mutations within melanoma-specific hotspots could influence tumour gene expression (termed “somatic eQTLs”).

**Fig. 4:**
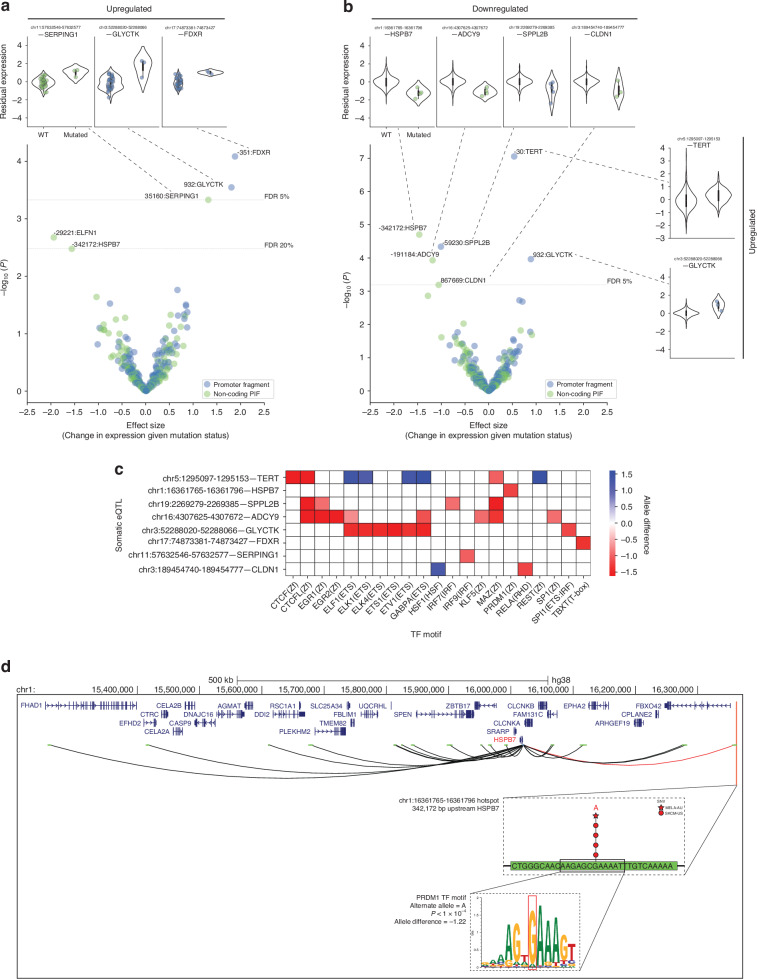
Eight melanoma somatic mutation hotspots are significantly associated with expression level changes of their Hi-C interacting target gene (FDR < 0.05, somatic eQTLs). **a** Melanoma-only analysis: three significant somatic eQTLs (FDR < 0.05), identified through multivariate linear regression, adjusting for CNA, promoter methylation, sex, and seven hidden factors. Expression is given as log_2_-transformed normalised RSEM value, z-score standardised across melanoma patients (n = 37). Normalisation involved dividing each gene’s raw RSEM count by the 75th percentile value within each patient and multiplying by 1000. **b** Extended analysis (22 cancer types): six significant somatic eQTLs (FDR < 0.05), still targeting melanoma hotspots. This analysis incorporates additional covariates for cancer type, ancestry, and 16 hidden factors. Expression is given as log_2_-transformed normalised RSEM value, z-score standardised across all cancer patients (n = 690). Blue and green colours in volcano plots indicate the fragment class encompassing each somatic eQTL. Somatic eQTLs in the volcano plots are labelled by the distance in base pairs relative to the start coordinate of the target gene in GENCODE v43 (adjusted for gene strand orientation). Violin plots detail the residual gene expression in WT vs. mutated patients for somatic eQTLs with FDR ≤ 5%. **c** Prediction of transcription factor (TF) binding sites disrupted or created by SNVs in somatic eQTLs significant at FDR ≤ 5% in the melanoma-only and/or extended analyses. The DNA binding motif of each TF is listed in parenthesis on the x-axis. Allele difference is the difference between the TF binding score on the WT and mutated allele. Negative and positive values indicate the disruption and creation of TF motif, respectively. **d** Chromatin looping interactions between the *HSPB7* promoter and distal non-coding promoter-interacting fragments. A somatic eQTL is predicted to down-regulate *HSPB7* expression by disrupting a PRDM1 TF binding motif.

Of the 107 patients in the melanoma PCAWG cohort with WGS data, 37 from the TCGA SKCM-US project had matching RNA-seq data available for this analysis. For each gene linked to at least one hotspot via our promoter-interaction network, we fit a multivariate linear regression model to evaluate its expression against the mutation status of its linked hotspot(s) and covariates such as copy number alteration (CNA), promoter methylation, sex, and 7 hidden factors [45] (methods) [Supplementary Table 7a]. This approach identified three hotspots as somatic eQTLs that upregulates three target genes at an FDR ≤ 0.05: *FDXR*, *GLYCTK*, and *SERPING1* [Fig. 4a]. Relaxing the threshold to FDR ≤ 0.2 identified two additional suggestive association with the downregulation of *HSPB7* and *ELFN1* [Fig. 4a].

Given the constraints of our small cohort size and the potential for pan-cancer eQTL signals, a comparative re-analysis was conducted using data from all 22 TCGA cancer types within PCAWG (690 TCGA individuals with WGS and matching RNA-seq data; still targeting melanoma hotspots). Adjustments were made for additional covariates, including cancer type, ancestry, and 16 hidden factors (methods) [Supplementary Table 7b]. This extended analysis identified six somatic eQTL-target gene pairs at an FDR of ≤ 0.05, with the somatic eQTL for *TERT* showing the most significant association, consistent with the pan-cancer *TERT* signal found earlier [8–10] [Fig. 4b]. Apart from *TERT*, all other somatic eQTL associations were melanoma-specific [Supplementary Table 7b]. The upregulated genes identified were *TERT* and *GLYCTK*, while the downregulated genes were *HSPB7*, *CLDN1*, *SPPL2B*, and *ADCY9*. Out of the five genes initially identified at an FDR ≤ 0.2 in the melanoma-only analysis, *GLYCTK* and *HSPB7* reached FDR ≤ 0.05 in the extended analysis [Fig. 4b]. All five had the same direction of eQTL effect in both analyses [Supplementary Table 7a, b].

One way that regulatory elements can modulate gene expression is by recruiting transcription factors (TFs) that bind to specific DNA sequence motifs [46]. Using motifbreakR [47], we assessed the impact of SNVs within somatic eQTLs on TF binding motifs (TFBMs). All eight significant somatic eQTLs (FDR ≤ 0.05 in the melanoma-only and/or extended analyses) were found to harbour SNVs that disrupt or create at least one TFBM [Fig. 4c, Supplementary Table 8]. Notably, five of these somatic eQTLs were found to reside within accessible chromatin regions in melanoma cells, as identified from DNase-seq and FAIRE-seq data [Supplementary Fig. 5]. To evaluate the functional relevance of these TFs, we analysed their expression levels and presence of predicted loss-of-function (pLoF) mutations. Other than TBXT, all identified TFs showed appreciable expression (median normalised RSEM > 1) across the SKCM-US melanoma cohort [Supplementary Fig. 6]. In the relevant patients harbouring TFBM-disrupting mutations, these TFs maintained normalised RSEM > 1 [Supplementary Fig. 6] and none harboured pLoF mutations [Supplementary Fig. 7], suggesting that they are likely functional in the patients where TFBM-disrupting mutations are present.

As an illustrative case, the G > A mutation within the eQTL at chr1:16361765-16361796 is predicted to strongly disrupt the binding affinity of the PRDM1 TF, thus potentially explaining its association with the downregulation of *HSPB7*, located 342,173 bp away [Fig. 4d]. Interestingly, this somatic eQTL showed no association with its nearest expressed gene, *FBXO42*, just 9286 bp away [Supplementary Fig. 8a]. None of the three other significant somatic eQTLs at non-coding PIF showed eQTL associations with their nearest genes [Supplementary Fig. 8b–d].

## Discussion

Identifying the full spectrum of genetic alterations driving melanoma is fundamental to understanding the molecular aetiology of melanoma and progressing towards more advanced, targeted therapies. Given the potential of non-coding mutations in altering gene regulatory mechanisms and melanoma development, our study aims to uncover hidden drivers in these regions. By leveraging melanoma-specific Hi-C data, we identified distal non-coding genomic fragments that interact physically with well-annotated promoters of 18,044 protein-coding genes. These fragments, termed “non-coding promoter-interacting fragments” (non-coding PIFs), form the basis of our approach, which, while conceptually similar to promoter-capture Hi-C (PCHi-C), leverages available Hi-C data to provide a viable framework for promoter-interaction analysis in the absence of melanoma-specific PCHi-C datasets. The non-coding PIFs are under stronger sequence constraint, enriched for histone marks of gene regulation, and harbour lower somatic mutation rate compared to their non-promoter-interacting counterparts (non-coding non-PIFs), making them suitable candidate regions for investigating driver mutations.

Using WGS and RNA-seq data from the PCAWG project, we discovered 102 and 304 hotspots harbouring recurrent SNVs in non-coding PIFs and promoters, respectively, within melanoma samples. Among these, 3 hotspots—1 in non-coding PIF and 2 in promoters—were identified as somatic eQTLs affecting target gene expressions in melanoma. Extending the analysis of the same hotspots across all 690 TCGA cancer patients within PCAWG identified 5 additional somatic eQTL associations. This relatively limited number of identified associations likely reflects our current limited statistical power. However, it is also possible that the influence of some hotspots may extend beyond transcriptional regulation. Additionally, despite our thorough adjustments for established general and melanoma-specific mutation rate covariates, it remains possible that some of the non-eQTL hotspots may be passenger or false positives.

Our study identified a total of eight target genes whose expression levels are altered by eight somatic eQTLs which contain SNVs that interfere with TF binding motifs. Aside from *TERT*, the other seven eQTLs are melanoma-specific, and their target genes have not been appreciably recognised as melanoma drivers. Nonetheless, most have been implicated as tumour suppressors or oncogenes in various cancer types. For example, *HSPB7*, a member of the small heat shock protein family, has been implicated as a tumour suppressor in renal cell carcinoma (RCC), where it is commonly downregulated [48]. Consequently, ectopic introduction of *HSPB7* suppressed RCC cancer cell lines growth [48]. Here, we found *HSPB7* as being downregulated by a somatic eQTL at non-coding PIF positioned 342,173 bp upstream. Furthermore, this somatic eQTL contain SNVs that strongly disrupt the binding site of PRDM1 TF. *PRDM1* is a tumour suppressor in melanoma [49] and other cancers [50–53], and loss of *PRDM1* has been shown to accelerate the onset and progression of melanoma [49].

Additionally, we identified *CLDN1* as being downregulated by a somatic eQTL in non-coding PIF positioned 867,699 bp downstream. Known for its role in cell adhesion, *CLDN1* is downregulated in metastatic melanomas compared to benign nevi [54]. The introduction of *CLDN1* into melanoma brain metastatic cells expressing low levels of *CLDN1* has been shown to suppress their metastatic phenotype [54]. *CLDN1* is also implicated as a tumour suppressor in lung adenocarcinoma [55], prostate [56] and oestrogen receptor-positive breast cancer [57]. Similarly, *ADCY9*, downregulated by a somatic eQTL in non-coding PIF positioned 191,184 bp upstream, has been recently identified as a novel tumour suppressor in lung adenocarcinoma [58]. Furthermore, we discovered a somatic eQTL in the *FDXR* promoter, associated with *FDXR* upregulation. Supporting this, a recent study has shown that *FDXR* drives the proliferation of primary and endocrine-resistant breast cancer cells by promoting mitochondrial fatty acid oxidation through *CPT1A* upregulation [59]. Importantly, *CPT1A* is a potential therapeutic target in melanoma, with its knockdown shown to inhibit the proliferation of V600E melanoma cells [60]. Thus, our study identified seven novel genes potentially driving melanoma, inviting deeper investigations to clarify their roles in melanoma.

Long-range *trans* associations are pivotal in elucidating the role of germline GWAS SNPs in complex diseases [26, 61–63], suggesting their potential significance in elucidating the impacts of somatic mutations in cancer. Challenges such as limited sample sizes have constrained the study of germline *trans*-eQTLs [64]. Given the power of detection and low sample sizes, our somatic eQTL analyses were only powered to detect *cis*-eQTLs. Therefore, all significant eQTL associations corresponded to *cis*-regulated genes. However, all four significant somatic eQTLs at non-coding PIF lacked associations with their nearest gene promoters, with three out of four targeting a gene located beyond 100,000 bp away. The most distant association was observed for *CLDN1*, with its regulating somatic eQTL positioned 867,669 bp downstream. Additionally, *MSR1* (a tumour suppressor in prostate cancer [65] and chronic myeloid leukaemia [66]) presented the most suggestive *trans* association, downregulated by a somatic eQTL positioned 1.5 Mb downstream (*P* = 0.009, FDR ≤ 0.3). These observations highlight the limitation of the nearest-gene approach in identifying true target genes, which overlooks the complexity of long-range gene regulatory interactions. Our approach, which identifies somatic eQTL associations within hotspots with confirmed physical interactions in melanoma cell lines, not only improves the accuracy in pinpointing their true tissue-specific target genes but also narrows down the search space, thus enhancing the power to detect somatic eQTLs.

The datasets used in this study present both strengths and limitations. Our use of melanoma-specific Hi-C datasets to construct a promoter-interaction network captures the tissue-specificity of 3D genome organisation [28]. Nonetheless, our current datasets do not address the dynamic [67] and potentially individual-specific nature of the 3D genome organisation, which extends beyond tissue-specificity. Furthermore, Hi-C, unlike PCHi-C, isn’t optimised for detecting promoter interactions and thus may not capture all interactions due to its lower effective sequence depth for these regions [68, 69]. Therefore, our analysis may overlook additional long-range interactions that a melanoma-specific PCHi-C could potentially reveal. Finally, our analysis is constrained by the limited expression data for melanoma, with only 37 matched WGS and RNA-seq datasets available. Despite these limitations, we have identified several melanoma-specific hotspots of somatic mutations affecting target gene expressions. Future studies that incorporate larger cohorts, examine distinct melanoma subtypes, such as acral melanoma [70], or other mutation types, such as indels, could offer deeper insights by revealing additional associations or signals specific to certain subtypes.

In conclusion, our study suggests additional non-coding drivers beyond the well-characterised *TERT* promoter in melanoma. These drivers are melanoma-specific, located in promoter-interacting DNA regions, alter transcription factor binding motifs, and affect the expression of genes previously implicated as tumour suppressors/oncogenes in various cancer types, highlighting the disruption of complex regulatory networks by non-coding mutations that may contribute to melanoma development. Furthermore, our study provides a framework for integrating multiple levels of biological data to uncover cancer-specific non-coding drivers, providing a solid foundation for targeted functional validations.

## Materials and methods

### In-silico digestion of the human genome

The hg38 human reference genome sequence was downloaded from NCBI (https://www.ncbi.nlm.nih.gov/datasets/genome/GCF_000001405.26/) and digested *in-silico* at HindIII restriction enzyme cleavage sites (A/AGCTT) into 851,637 non-overlapping genomic fragments using the Restriction package of Biopython [71].

### Defining gene promoters

Data on active transcription start sites (TSSs) (i.e., refTSS_v3.3_human_coordinate.hg38.‌‌bed and refTSS_v3.3_human_annotation.txt) was obtained from refTSS v3.3 [30] (https://reftss.riken.jp/datafiles/3.3/human/). Each TSS was mapped to one protein-coding gene annotated by GENCODE v43 [72], and TSSs located on chrM and chrY were excluded.

For each TSS mapped to the same gene, a 2000 bp upstream and 200 bp downstream extension (relative to the DNA strand of the mapped gene) was performed, followed by merging of any overlapping intervals. The resulting interval(s) define the gene’s promoter region(s). The pybedtools [73] python library was used to identify the genomic fragment(s) encompassing the gene’s promoter region(s), thus defining the “promoter fragment(s)” of that gene. In total, we identified the promoter fragments of 18,044 protein-coding genes located within 41,733 unique genomic fragments.

### Construction of a melanoma promoter-interaction network

As the spatial interactions of genomes are tissue-specific [28, 29], we focused on analysing Hi-C chromatin interaction libraries from two melanoma cell lines: SK-MEL-5 and RPMI-7951 (two replicates each). Raw Hi-C data were downloaded from GEO (https://www.ncbi.nlm.nih.gov/geo/, accession: GSE105491 and GSE106022 for SK-MEL-5 and RPMI-7951, respectively) and processed (as previously described [74]) to obtain Hi-C chromatin interaction library files with the following format: read name, strand1, chr1, position1, fragment1, strand2, chr2, position2, fragment2. Each row in the processed interaction files describes the alignment of the two interacting read pairs (i.e., 1 and 2). Our pipeline works with any Hi-C pipeline that can generate interaction files in the required data format (e.g., HOMER [75], Juicer [76]).

To characterise the regulatory landscape of melanoma, we mapped the genome-wide physical interactions anchored to the promoter fragment(s) identified for each gene. These promoter interactions were identified on a presence/absence basis. A fragment-promoter interaction is valid whenever it has ≥2 supporting interactions from ≥2 different replicates of ≥1 cell lines. The resulting promoter-interaction network [Supplementary File 1] identified 459,862 non-promoter distal fragments (promoter-interacting fragments; PIFs) that interacted with at least one gene promoter, making 1,321,059 unique interactions.

Overall, this approach allowed for the classification of the total genomic fragments (n = 851,637) into distinct fragment classes:

1. Promoter fragment (n = 41,733)
2. PIF: exon-overlapping (n = 84,353) or non-coding (n = 375,509)
3. Non-PIF: exon-overlapping (n = 13,379) or non-coding (n = 336,663)

### Sequence constraint analysis

We downloaded the raw depletion rank (DR) score data from the supplementary data of Halldorsson et al. [33] This dataset (i.e., DR.gor) contains the associated DR score for each overlapping 500-bp windows in the genome with a 50-bp step size. The pybedtools Python library was used to identify every 500-bp window that had an overlap of more than 50% with each of the 851,637 genomic fragment. The median DR score for each fragment was then calculated and assigned as the corresponding DR score for that fragment. Statistical significance was determined using Wilcoxon rank-sum test with Bonferroni correction for multiple comparisons.

### Histone mark enrichment analysis

We downloaded narrowpeak ChIP-seq data on 6 histone marks (H3K4me1, H3K27ac, H3K4me3, H3K27me3, H3K36me3 and H3K9me3) from 127 tissues and cell lines from Roadmap Epigenomics [77] (https://egg2.wustl.edu/roadmap/data/byFileType/peaks/consolidated/narrowPeak/). All genomic coordinates were converted to hg38 using LiftOver [78] tool. These data were then overlapped with the non-coding PIFs that we identified. We selected random non-coding genomic fragments that matched the total length of the identified non-coding PIFs. We performed resampling of the random non-coding genomic fragments 1000 times and assessed the statistical significance of the enrichment using permutation test. Fold changes were calculated by dividing the rate of overlap of ChIP-seq peaks in non-coding PIFs and in non-coding non-PIFs, and subsequently, log_2_ transformed.

### Identification of somatic mutation hotspots

The PCAWG SNV/INDEL concensus callsets of ICGC (i.e., final_consensus_snv_indel_ passonly_ icgc.open.tgz) and TCGA (i.e., final_consensus_snv_indel_tcga. controlled.tgz) were downloaded from the ICGC portal (https://dcc.icgc.org/releases/PCAWG/consensus_snv_indel/). All genomic coordinates were converted to hg38 using LiftOver tool. MutSpot [36] was then used to identify focal genomic regions (hotspots) harbouring recurrent SNVs identified from 70 ICGC and 37 TCGA melanoma patients. MutSpot was run separately on promoter fragments and non-coding PIFs by specifying the *region.of.interest* option. Additionally, the following options were specified:

1. The LASSO stability threshold, *cutoff.nucleotide* and *cutoff.features*, was set at 1 and 0.98, respectively.
2. The window size for hotspot discovery, *hotspot.size*, was set to 31 bp.
3. Minimum number of mutated samples in each hotspot, *min.count*, was set to 4.

MutSpot provides a default pool of 135 features as potential covariates in the background mutation model calculation. As suggested [36], we added the following additional features to correct for known melanoma-specific mutational biases:

1. Narrowpeak ChIP-seq on 6 histone marks (H3K4me1, H3K27ac, H3K4me3, H3K27me3, H3K36me3 and H3K9me3) and DNase-seq data from melanocytes to correct for melanocyte-specific local variations in mutation rate (downloaded from Roadmap Epigenomics as described earlier).
2. Intervals of CTTCCG motif to correct for known vulnerability to UV-induced mutagenesis at these sites [37].
3. Intervals of transcription factor binding sites in melanoma cell line to correct for known hypermutations due to impaired nucleotide excision repair at these sites [38] (downloaded from http://bg.upf.edu/group/projects/tfbs/).

Significant hotspots were determined using an FDR cutoff of ≤ 0.05. Hotspots at fragment boundaries were excluded from further analyses.

### Somatic eQTL data processing: RNA-seq, CNA, promoter methylation, and other covariates

Normalised RSEM RNA-seq data (i.e., *.rnaseqv2__illuminahiseq_rnaseqv2__unc_edu__Level_3__ RSEM_genes_normalized__data.data.txt) were downloaded from GDAC firehose (https://gdac.broadinstitute.org). This normalisation involved dividing the raw RSEM count of each gene by the 75^th^ percentile value within the corresponding patient and multiplying by a factor of 1000. Genes were selected for further analysis if their normalised RSEM values exhibited a median of >1 across all patients, resulting in 16,109 and 16,604 expressed genes in the melanoma-only and extended analysis, respectively. Normalised RSEM values were further log_2_ transformed and z-score standardised.

Hidden factors influencing gene expression were identified using probabilistic estimation of expression residuals (PEER) [45]. For the melanoma-specific and extended analyses, we estimated 20 and 50 potential hidden factors, respectively, incorporating a mean term and covariates sex, ancestry, and cancer type during estimation. Information on sex, ancestry, and cancer type for each patient was obtained from Supplementary Table 1 of the main PCAWG publication [79]. Analyses of the posterior variance of factor weights suggested 7 and 16 optimal hidden factors for the melanoma-only and extended analysis, respectively.

PCAWG consensus callset for CNA (i.e., all_samples.consensus_CN.by_gene.170214.txt) was downloaded from the ICGC portal (https://dcc.icgc.org/releases/PCAWG/consensus_cnv/gene_level_calls). Genes with missing CNA in at least one patient were removed from further analysis.

DNA methylation data from the Illumina HumanMethylation450 BeadChip array (i.e., *.methylation__humanmethylation450__jhu_usc_edu__Level_3__within_bioassay_data_set_function__data.data.txt) were downloaded from GDAC firehose (https://gdac.broadinstitute.org). For each patient, the methylation value of each gene was calculated as the mean beta value of probes located within the gene’s promoter region, as defined in the “Defining gene promoters” section. Among the 690 TCGA patients within PCAWG with both WGS and RNA-seq data available, 537 (including all SKCM-US patients) had methylation data available. For the remaining patients, methylation values were imputed using the mean methylation values for each gene across patients. Genes with missing methylation data in at least one patient were removed from further analysis.

For the somatic eQTL analyses, only hotspots that are mutated in ≥ 3 SKCM-US patients were considered. Ultimately, 290 and 297 genes linked to at least one hotspot, expressed, and had CNA and methylation data were selected for somatic eQTL calling in the melanoma-only and extended analysis, respectively.

### Somatic eQTL analysis

For each selected gene, we fit a multivariate linear regression model to evaluate its expression against the mutation status of its linked hotspot(s) and other covariates as follows:

$${ex}{p}_{g}= 	 \ {\beta }_{0}+{\beta }_{h1}h1+\ldots +{\beta }_{{hn}}{hn}+{\beta }_{{CNA}}{CNA}+{{{\beta }}}_{m}m+{\beta }_{{sex}}{sex}\\ 	 +{\beta }_{{anc}}{anc}+{\beta }_{{CT}}{CT}+{\beta }_{f1}f1+\ldots +{\beta }_{{fn}}{fn}$$

Where *exp*_*g*_ represent the normalised gene expression level *exp* of gene *g* and *β* values represent the regression coefficients for:

- The mutation status of its linked hotspot(s), *h* (0=wild-type, 1=mutated)
- Copy number alteration levels, *CNA* (0=neutral, 1=amplified, 2=highly amplified, −1=deleted, -2=deeply deleted)
- Promoter methylation, *m* (mean beta values)
- Sex, *sex* (0=female, 1=male)
- Ancestry, *anc* (5 ancestries: European/African/East Asian/Admixed American/South Asian, one-hot encoded)
- Cancer type, *CT* (22 cancer types, one-hot encoded)
- Hidden factors, *f* (real values)

For the melanoma-only analysis, the covariates considered were *h*, *sex*, *CNA*, *m*, and *f* because this cohort is homogeneous with respect to *CT* and consisted exclusively of individuals of European *anc*. The extended analysis, however, incorporated all covariates.

To address the high dimensionality of the data, we used Lasso for feature selection by adding an L1-norm to the objective function, minimising the squared error between actual and predicted gene expression levels for each gene:

$${\left(e-\hat{e}\right)}^{2}+\lambda {{{\rm{||}}}}\beta {{{\rm{||}}}}^{1}$$

The regularisation parameter *λ* was optimised through 10-fold cross-validation. In cases where the L1-norm led to all *β*_*h*_ coefficients being zero, *λ* was decreased to include at least one hotspot. This step ensures that we can always evaluate the effect of hotspots on gene expression in the subsequent multivariate linear regression analysis. Importantly, all significant somatic eQTLs identified in the melanoma-only and/or extended analyses did not require such reduction in λ.

Once λ was optimised, the multivariate linear regression model was fitted using the selected feature set. We then derived a gene-level and individual hotspot-gene *P* values (some genes are linked to >1 hotspots). For the gene-level *P* value, we used F-statistics by comparing the full model accuracy against that of the simpler model without any hotspots. Individual hotspot-gene *P* value and effect size was obtained directly from the regression model. Multiple hypothesis was corrected using the Benjamini-Hochberg method independently for gene-level and individual hotspot-gene *P* values.

This approach for identifying somatic eQTLs involves using Lasso for feature selection, followed by fitting a multivariate linear regression model on the selected feature set to obtain *P* values. It does not include specific post-selection inference procedures, aligning with methodologies used in previous studies by Zhang et al. [80] and Soltis et al. [81].

### Transcription factor (TF) binding analysis

We used motifbreakR (v2.16.0) [47] to predict the impact of SNVs on TF bindings, utilising position weight matrices of 247 human TFs from the HOMER [75] motif data source provided by MotifDb (v1.42.0) [82]. This analysis focused on SNVs within the eight significant (FDR ≤ 0.05) somatic eQTLs in the melanoma-only and/or extended analyses. We used the “information content” scoring algorithm and applied a threshold of *P* < 1 × 10^−4^ to determine a motif match. motifbreakR classifies the normalised binding score difference between the reference and the alternate allele of each SNV as “neutral” (allele difference <0.4), “weak” (allele difference < 0.7), or “strong” (allele difference ≥0.7). We only consider strongly affected motifs.

### Open chromatin region analysis

To determine whether the identified somatic eQTLs reside within putative accessible chromatin regions, we performed an overlap analysis using DNase-seq and FAIRE-seq data from melanoma cultures. DNase-seq peaks were downloaded from the ENCODE portal (https://www.encodeproject.org) for SK-MEL-5 (file accessions: ENCFF453QYK and ENCFF211IRI) and RPMI-7951 (file accessions: ENCFF271CVJ and ENCFF736FKW). FAIRE-seq peaks from 11 individuals were downloaded from Verfaillie et al. [83] and converted to hg38 using LiftOver tool. Overlaps between somatic eQTL coordinates and DNase-seq or FAIRE-seq peaks were performed using pybedtools.

## Supplementary information

Supplementary Figures

Supplementary Tables

Supplementary File 1

## Data Availability

Access to the controlled SNV/INDEL consensus callsets of TCGA was approved for General Research Use by the dbGaP (https://www.ncbi.nlm.nih.gov/gap/) Data Access Committee (Project ID: 34995, accession: phs000178). The “Promoter Interaction Network” software used to construct and characterise melanoma promoter-interaction network is available on GitHub (https://github.com/MichaelPudjihartono/Promoter-Interaction-Network). The “regionperm” software used to assess the significance of histone mark enrichments through permutation tests is available on GitHub (https://github.com/MichaelPudjihartono/regionperm). Data analyses and visualisations were performed using Python (version 3.8.12) through Jupyter Notebook (version 6.4.6) or using R (version 4.0.4) through RStudio (version 1.4.1106). Additional in-house scripts used for data wrangling are available upon request.
