# Supplementary material for: Melanoma-specific mutation hotspots in distal, non-coding, promoter-interacting regions implicate novel candidate driver genes
Source: Br J Cancer. 2024 Oct 4;131(10):1644–55. doi: 10.1038/s41416-024-02870-w (PMC11555344; doi:10.1038/s41416-024-02870-w)
Supplement: Supplementary file 1 — Supplementary Figures [file 41416_2024_2870_MOESM1_ESM.pdf]

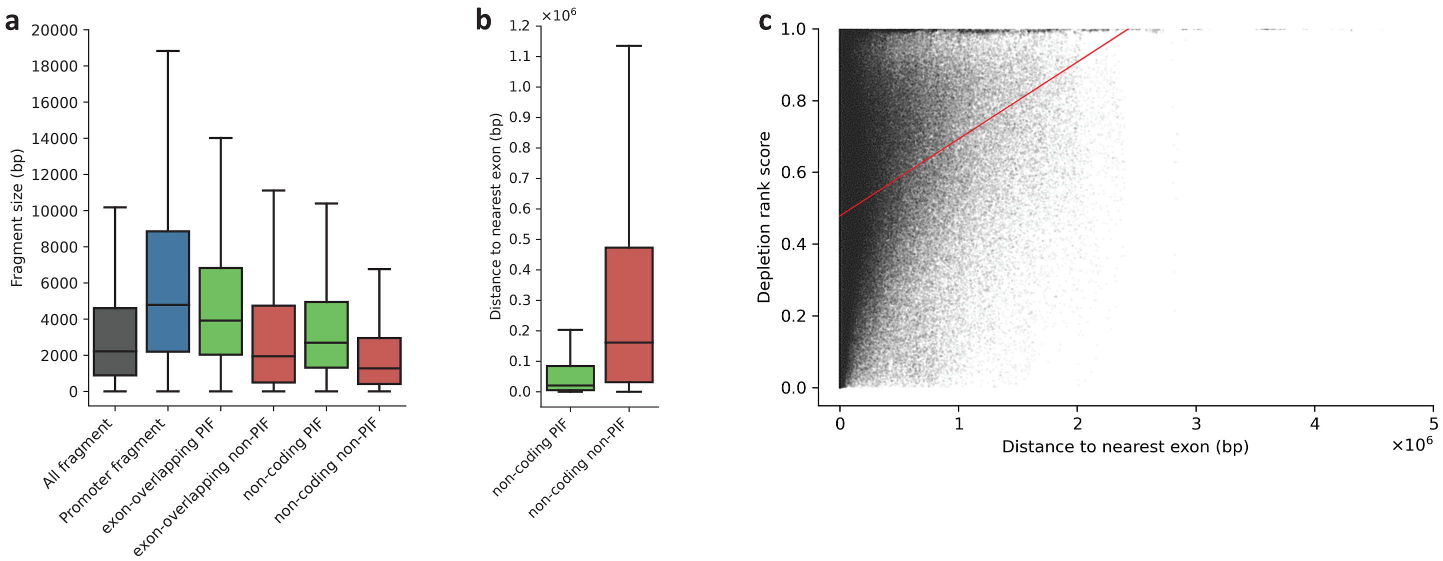

Supplementary Figure 1. Characteristics of genomic fragment classes. (a) Distribution of fragment sizes across different fragment classes. (b) Non-coding promoter-interacting fragments (PIFs) are located in closer proximity to coding exons compared to non-coding non-PIFs. (c) Depletion rank score of each individual fragment plotted against the distance to the nearest coding exon. Red line indicates the linear regression line of best fit.

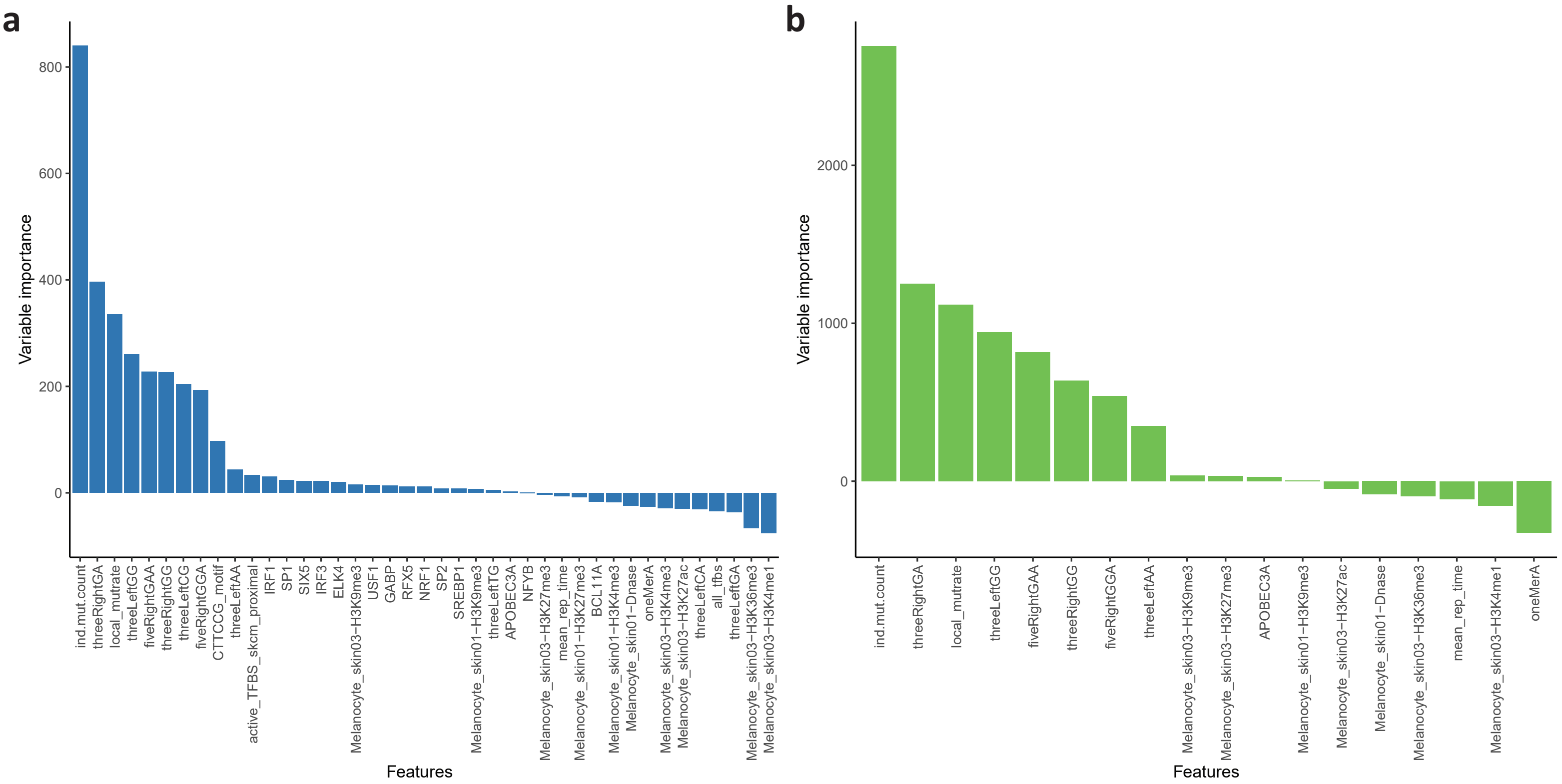

Supplementary Figure 2. Bar plot of feature importance of the background mutation model outputted by MutSpot for (a) promoter fragments and (b) non-coding promoter-interacting fragments.

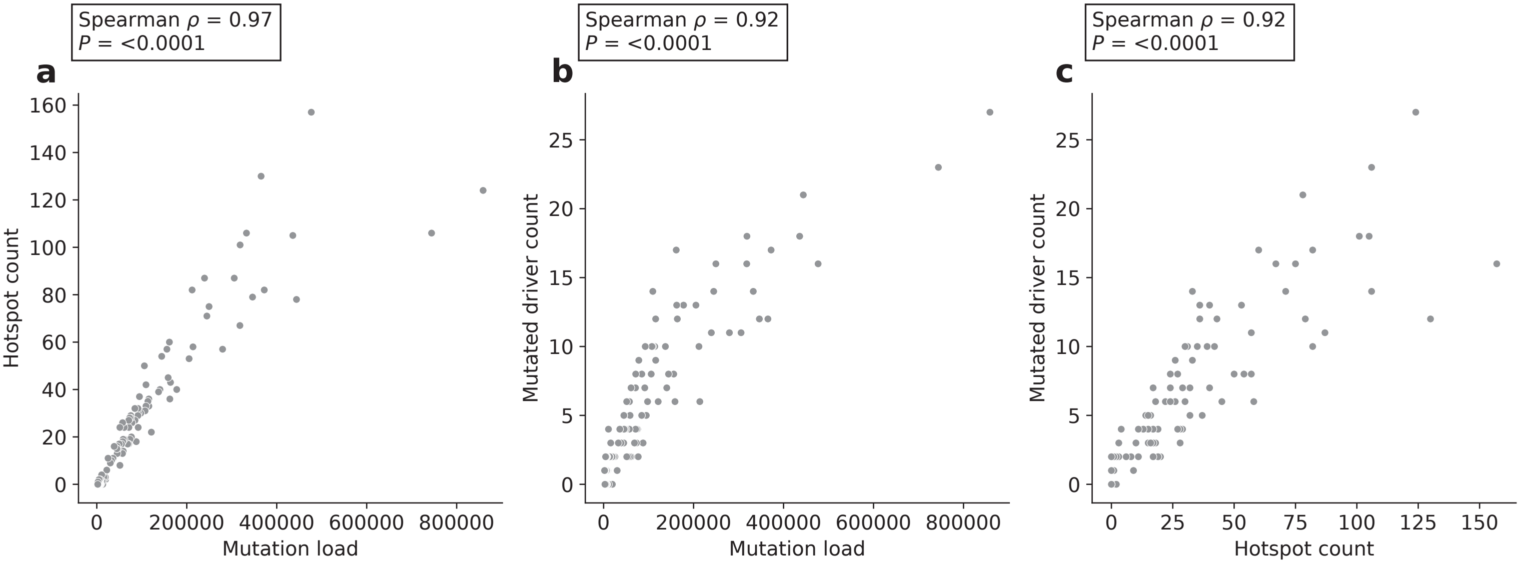

Supplementary Figure 3. Correlations between mutation load, hotspot count, and mutated driver gene count across 107 melanoma patients. The figure illustrates the correlation between (a) mutation load and hotspot count, (b) mutation load and mutated driver gene count, and (c) hotspot count and mutated driver gene count across patients. Each point represents a patient. The Spearman correlation coefficient (ρ) and *P* values (*P*) are shown above each plot, indicating strong positive correlations in each case. The list of established melanoma driver genes (n = 56) are obtained from the Cancer Gene Census.

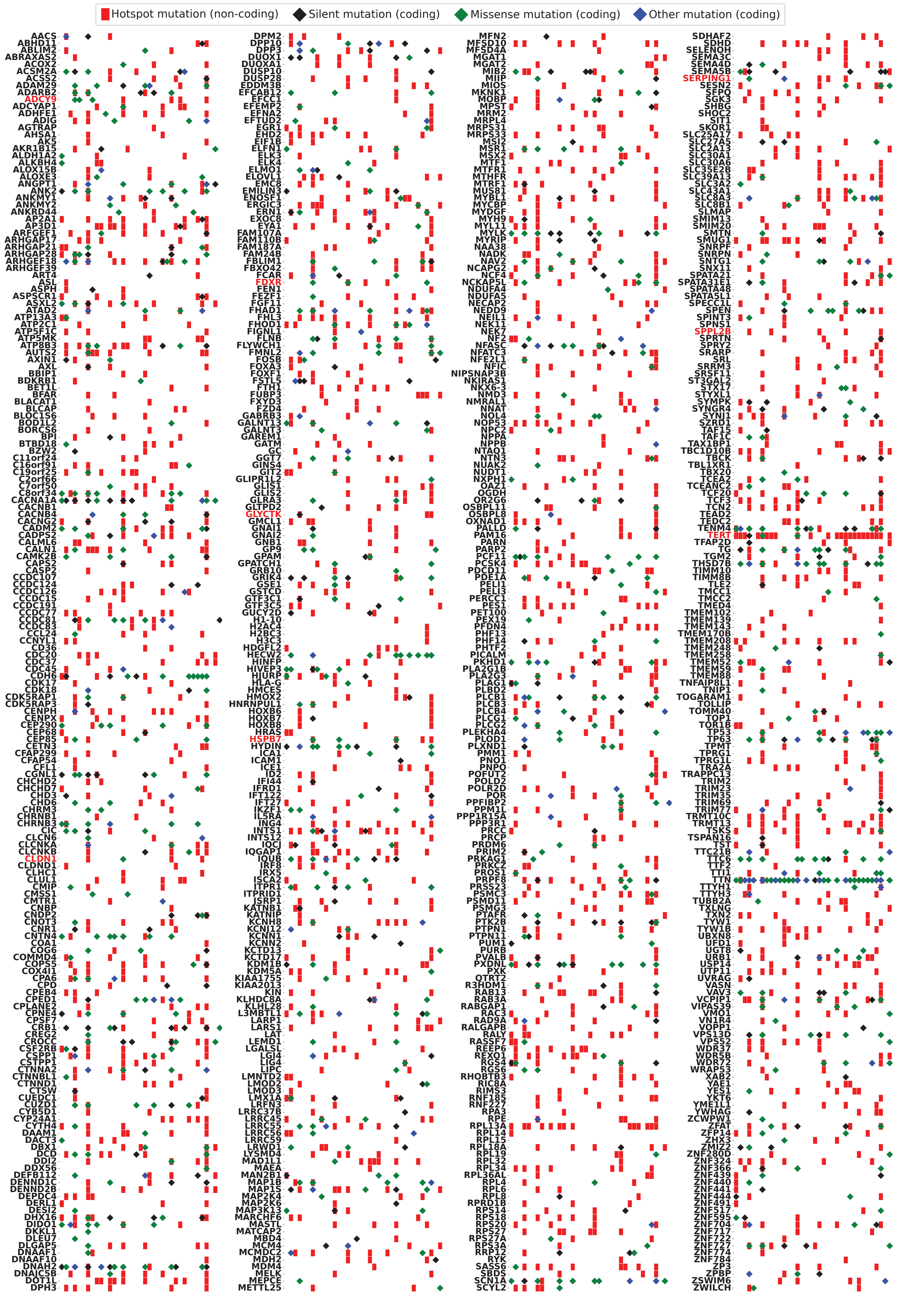

Supplementary Figure 4. Hotspot and coding mutation status of target genes in the SKCM-US melanoma cohort. The y-axis represents the gene names, and the x-axis represents individual patient (n = 37). A diamond indicates the presence of a coding mutation, while a red square denotes a mutation in the associated hotspot(s). Coding mutations are color-coded as follows: black for silent mutations, green for missense mutations, and blue for other coding mutations. Other coding mutations include nonsense mutation, splice site mutation, frame shift insertion, frame shift deletion, in-frame insertion, in-frame deletion, de novo start in-frame, de novo start out-of-frame, nonstop mutation, start codon insertion, start codon deletion, start codon SNV, stop codon insertion, and stop codon deletion. If multiple mutation types are present, the most impactful mutation type determines the color: missense mutations override silent mutations, and other coding mutations override both silent and missense mutations. Genes highlighted in red are targets of significant (FDR ≤ 5%) somatic eQTLs in the melanoma-only and/or extended analyses [Figure 4a and b].

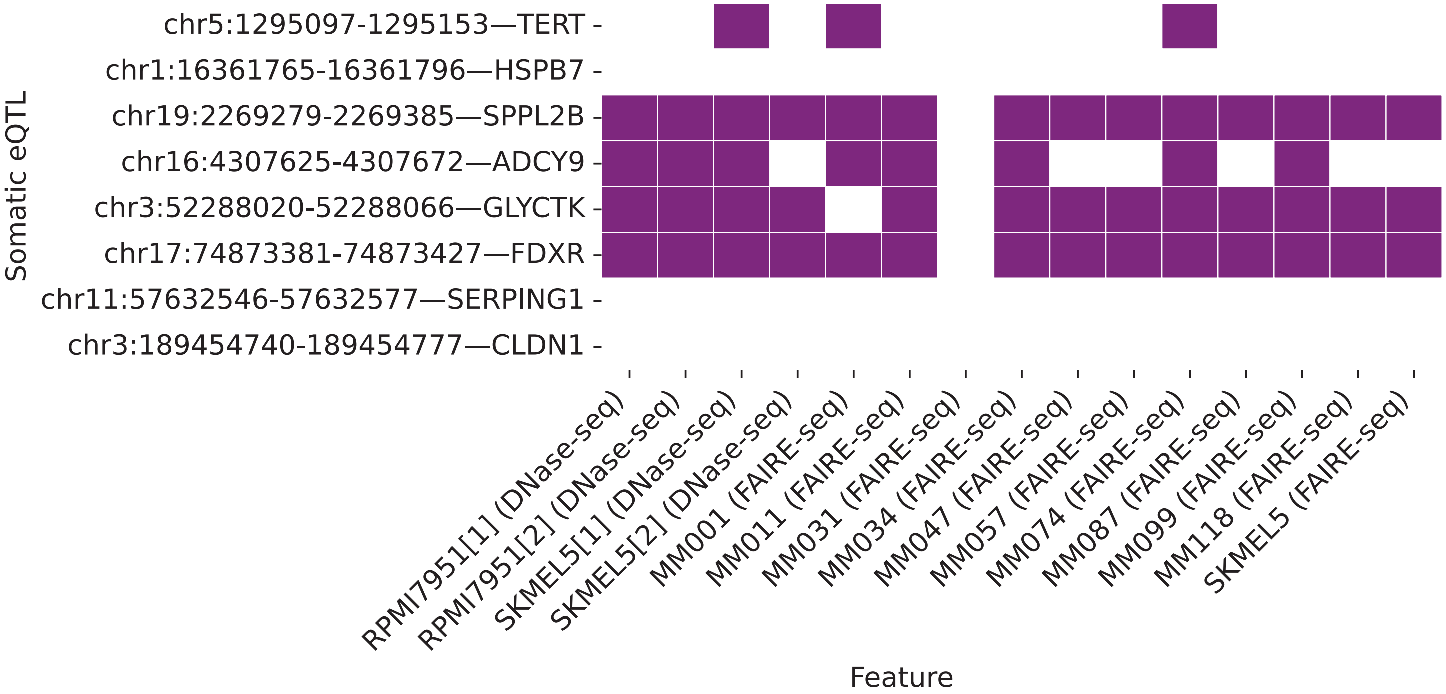

Supplementary Figure 5. Chromatin accessibility of somatic eQTLs in melanoma cells. Each purple square indicates an overlap between a DNase-seq or FAIRE-seq peak and the corresponding somatic eQTL coordinates. Square brackets denote isogenic replicates from the ENCODE project.

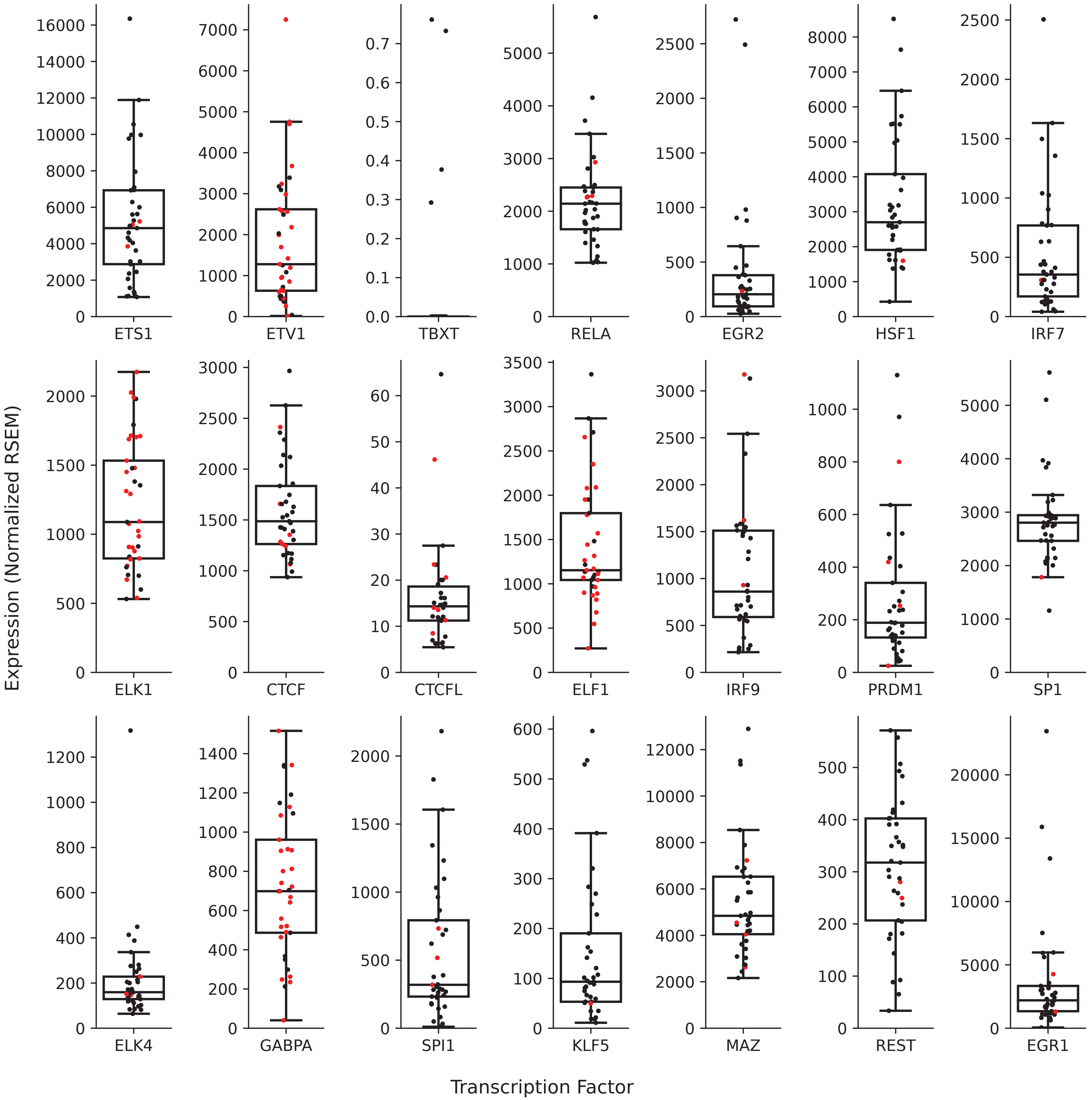

Supplementary Figure 6. Expression levels of 21 transcription factors (TFs) whose binding motif are disrupted by SNVs within somatic eQTLs. Expression is given as normalized RSEM value. Normalization involved dividing each gene's raw RSEM count by the 75th percentile value within each patient and multiplying by 1000. Each dot represents an individual patient, with those harboring TF binding motif-disrupting mutations within somatic eQTLs colored in red.

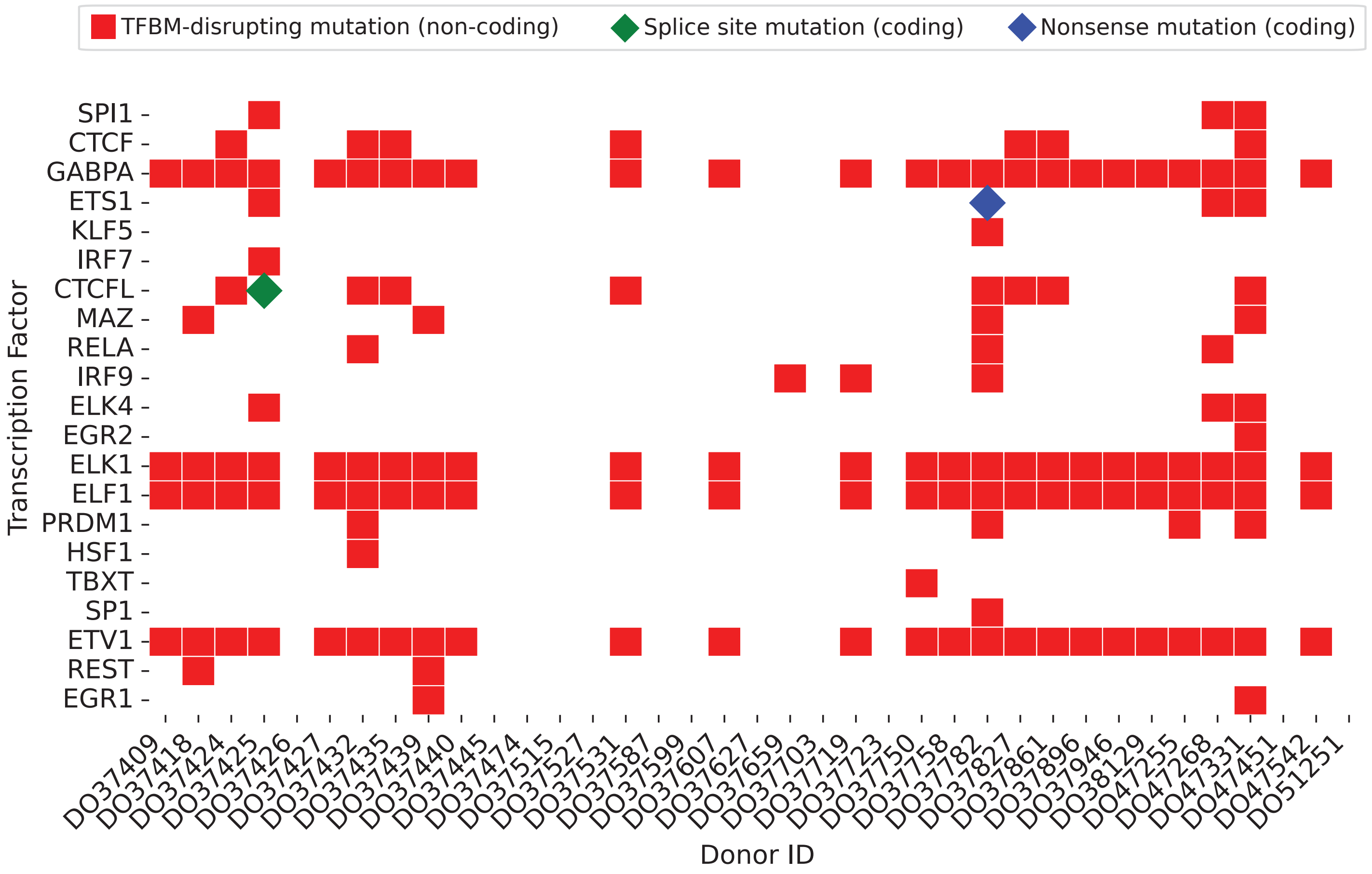

Supplementary Figure 7. Mutation profiles of 21 transcription factors (TFs) whose binding motifs are disrupted by SNVs within somatic eQTLs. The y-axis represents TFs, and the x-axis represents individual patient (n = 37). A red square denotes the presence of a TF binding motif-disrupting mutation within somatic eQTLs. A diamond indicates the presence of a predicted loss-of-functon(pLoF) mutation within the TF. pLoF mutations are defined as mutations that introduce a premature stop codon (nonsense mutation), shift-reported transcriptional frame (frameshift mutation), or alter splice site nucleotides (splice site mutation).

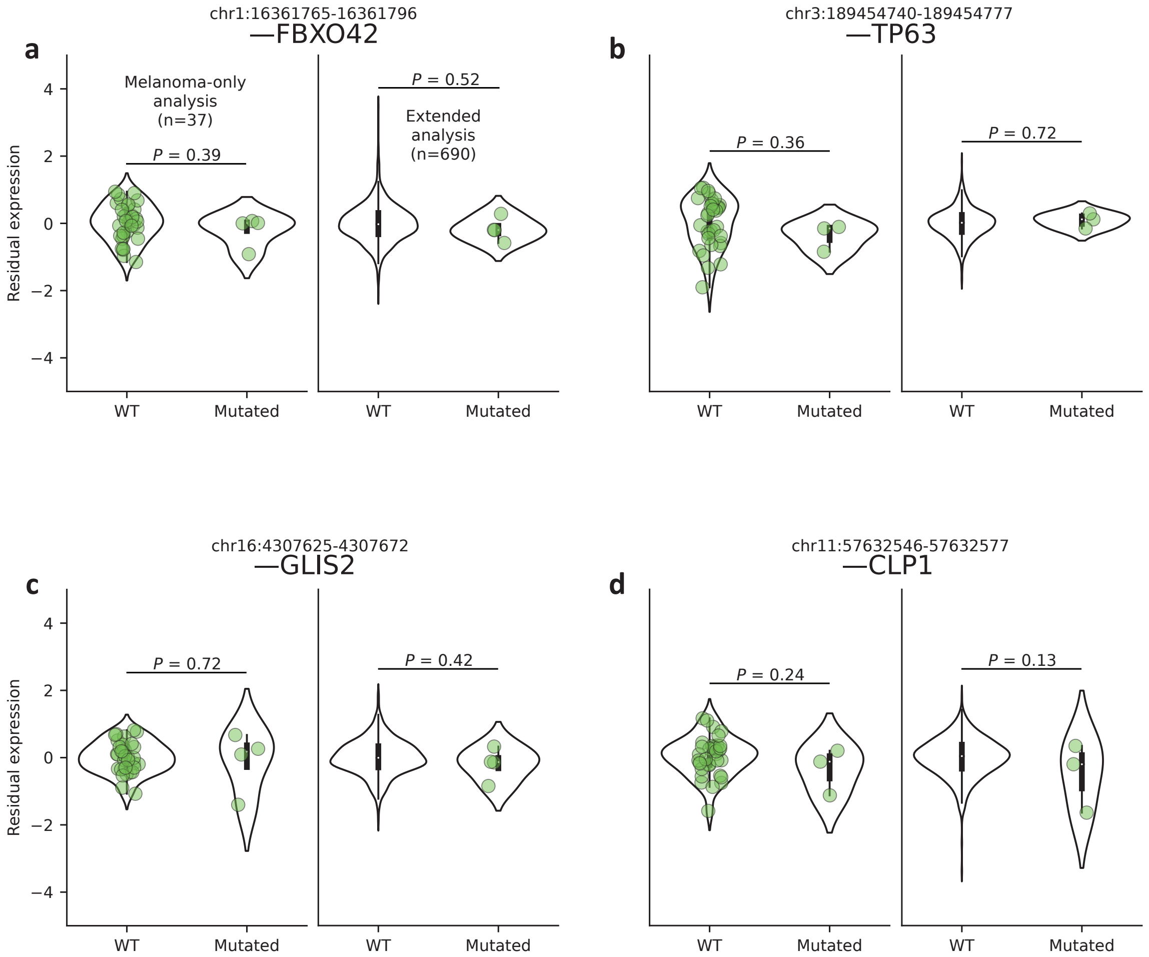

Supplementary Figure 8. Significant somatic eQTLs at non-coding promoter-interacting fragments lacked associations with the nearest gene based on proximity to the promoter. The violin plots compare residual gene expression levels between wild-type (WT) and mutated patients, specifically highlighting the hotspots (a) chr1:16361765-16361796 (somatic eQTL to *HSPB7*) and its nearest gene, (b) chr3:189454740-189454777 (somatic eQTL to *CLDN1*) and its nearest gene, (c) chr16:4307625-4307672 (somatic eQTL to *ADCY9*) and its nearest gene, and (d) chr11:57632546-57632577 (somatic eQTL to *SERPING1*) and its nearest gene.
